# Right Diaphragmatic Eventration with an Intrathoracic Kidney: Case Report and Review of the Literature

**DOI:** 10.1155/2018/2631391

**Published:** 2018-12-06

**Authors:** Anibal Carrasco, Ricardo Castro

**Affiliations:** Department of General Surgery, Jorge Reategui Delgado Hospital, Av. Grau block 11 s/n, Piura, Peru

## Abstract

We aimed to review the publications on the diagnosis of diaphragmatic eventration and report on the clinical presentation and surgical treatment of a female patient aged 17 years. The present case, though quite infrequent, shows the presence of an ectopic thoracic kidney on the right side. The clinical features included dry cough, chest pain, respiratory distress, and bronchial spasms for 4 years; additionally, the patient had episodes of bronchial asthma since her childhood. The right hemithorax presented invasion of the thin loops, right colon, and kidney. The treatment approach was laparoscopic followed by thoracotomy using a dual mesh. The intrathoracic kidney remained in place. The outcome was satisfactory. Diaphragmatic eventration associated with thoracic renal ectopia is a very rare entity, considering the age, sex, and right location of the condition. It represents a clinical and diagnostic challenge; clinicians, radiologists, and surgeons must be alert with a high degree of suspicion in order to correlate symptoms and imaging findings and understand the etiopathogenesis. In addition, they should plan an adequate and individualized surgical repair making use, as far as possible, of the minimally invasive procedures that are currently used.

## 1. Introduction

There are four types of congenital diaphragmatic defects: posterolateral Bochdalek diaphragmatic hernia, Morgagni-Larrey parasternal diaphragmatic hernia, peritoneal-pericardial diaphragmatic hernia, and diaphragmatic eventration [1]. The latter is also an acquired condition due to phrenic nerve injury (paralysis or paresis). It consists of the elevation of one, or less likely, both hemidiaphragms causing protrusion of the intra-abdominal viscera to the affected hemithorax. The hemidiaphragm may have a normal appearance or variable degree of tissue degeneration, thus forming a translucent membrane without muscle fibers [2]. The difference between diaphragmatic hernia and eventration is important; in the latter, there is no true defect. The incidence of diaphragmatic eventration is <0.05% [3]. The treatment must restore an active and effective contraction and improve the respiratory activity. This is achieved with diaphragmatic plication and can be performed via thoracotomy or using a minimally invasive laparoscopic or thoracoscopic route, with or without the interposition of a prosthetic material [4–6].

Thoracic renal ectopia is rare, with a prevalence of 1 in 10,000 cases of ectopic kidneys. There are about 200 published cases, mainly in male adults with the involvement of the left kidney [7, 8]. These are discovered incidentally with imaging studies. These are usually asymptomatic, with normal renal anatomy, and do not require any intervention. The embryological cause is related to accelerated ascent of the kidney to the upper region before closing the diaphragm with delayed closure of the pleuroperitoneal membrane. It is also believed that alterations in the development of adrenal glands and the liver can vary the position of the kidney [9, 10]. However, none of these mechanisms is completely clear.

We consider it important to report this case due to the inaccurate diagnosis of diaphragmatic eventration, owing to the coexistence of an intrathoracic ectopic kidney, location of the defect on the right side, sex of the patient, and the age of the patient at the time of diagnosis. Thus, it becomes necessary to increase the awareness on the need to individualize the treatment approach for this condition due to the low frequency of its pathology.

## 2. Case Presentation

**Figure 1:**
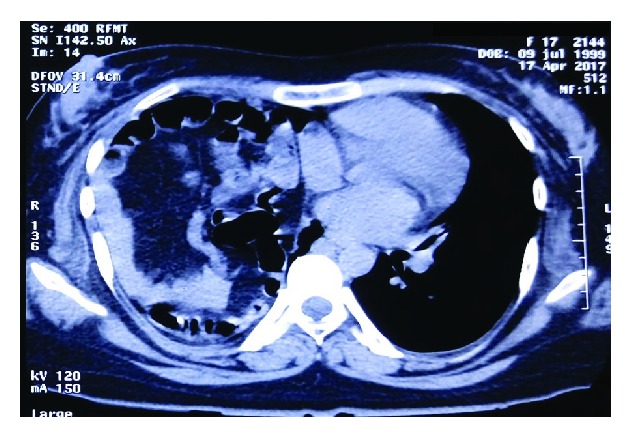
Axial section of both hemithoraces. Bowel loops occupying the right hemithorax.

**Figure 2:**
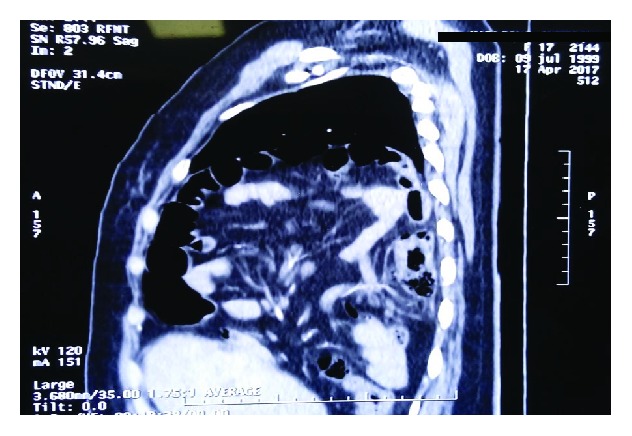
Sagittal section of the right hemithorax showing occupation by intestinal loops and mesentery.

**Figure 3:**
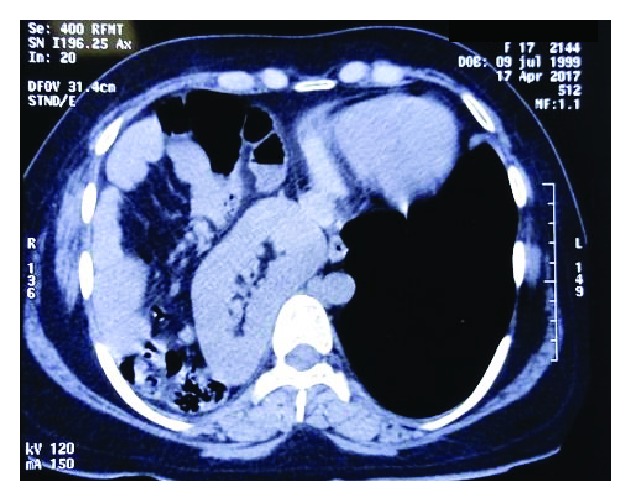
Axial section with the kidney in a posterior position and mild mediastinal compression.

**Figure 4:**
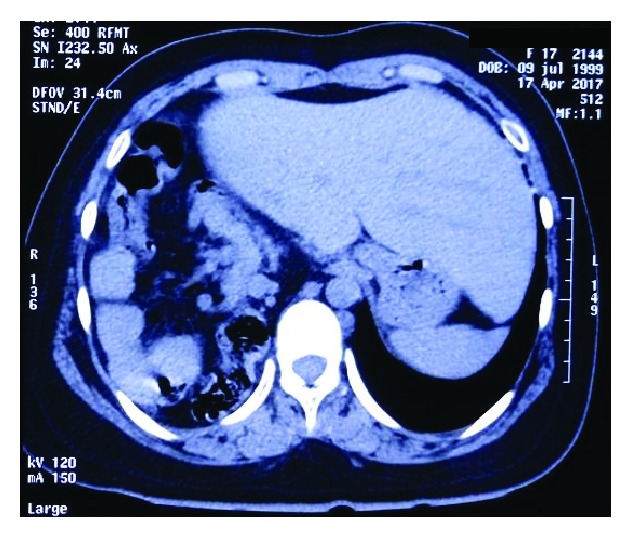
Notable hepatic displacement towards the left hemiabdomen that causes gastric compression.

**Figure 5:**
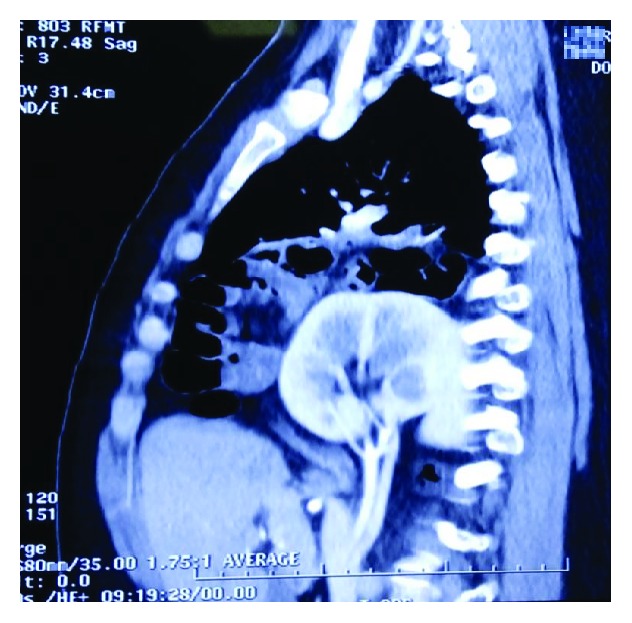
Sagittal section showing the occupation in a posterior situation of the kidney with the pedicle in the right hemithorax.

**Figure 6:**
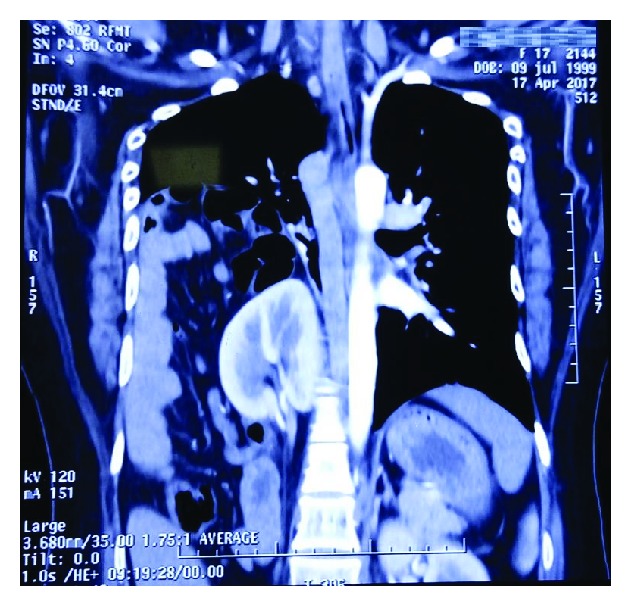
Contrast coronal section showing both hemithoraces. Lung parenchyma collapsed in the right hemithorax and occupied by the small intestine, right colon, mesentery, right kidney, and its corresponding pedicle. Normal left hemithorax.

We describe the case of a 17-year-old girl with dry cough, chest pain, respiratory distress, and bronchial spasms for 4 years and presumable repeated episodes of bronchial asthma since her childhood. Computed tomography showed the intestine, mesentery, colon, and kidney inside the right hemithorax (Figures 1–6); uniform and continuous diaphragmatic elevation throughout the hemithorax (Figures 5 and 6); discrete mediastinal displacement caused by the right kidney (Figure 3); and hepatic displacement towards the left hemiabdomen causing gastric compression (Figure 4). The right lung was found to be collapsed.

**Figure 7:**
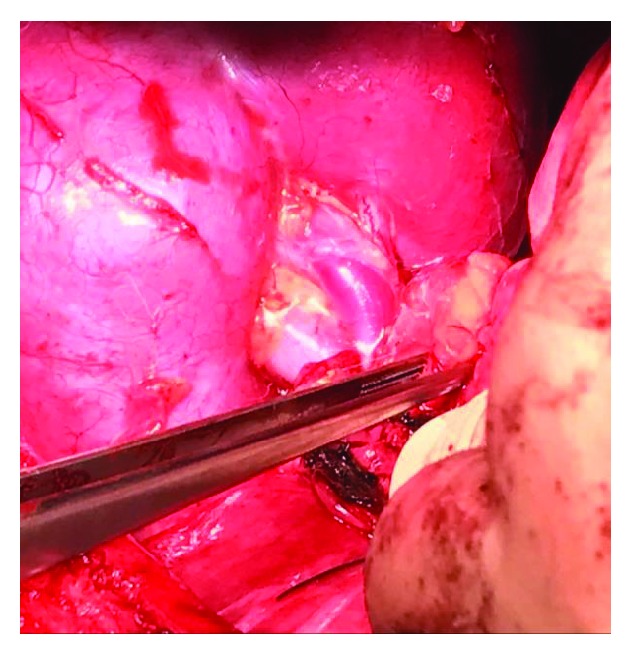
The right kidney and its respective pedicle in the posterior intrathoracic situation.

**Figure 8:**
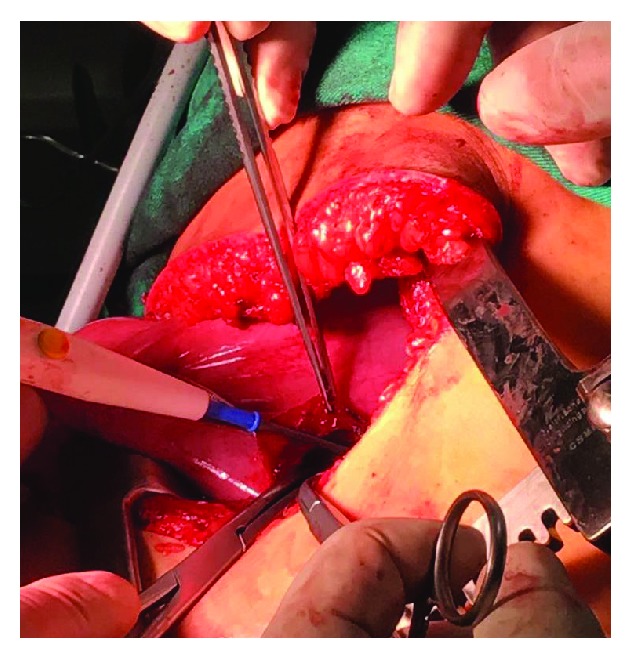
Incision of the laminated and elongated hemidiaphragm with the presence of muscle fibers.

**Figure 9:**
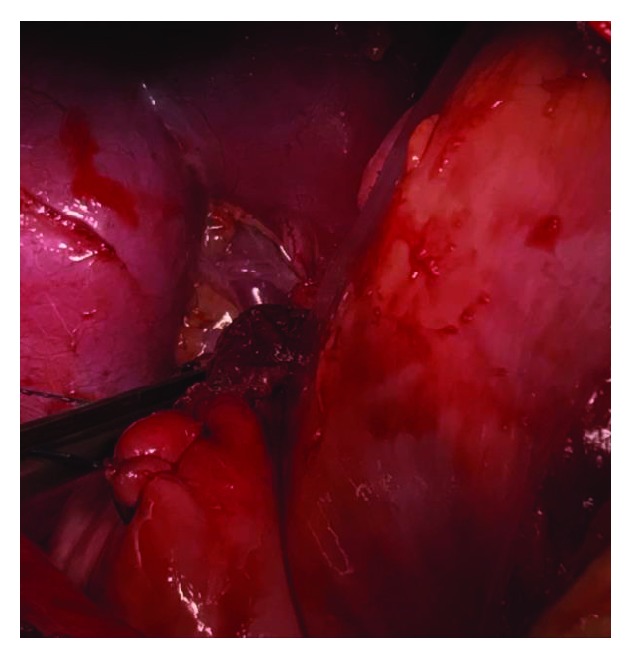
Suture of the hemidiaphragmatic defect. The kidney in the posterior view.

**Figure 10:**
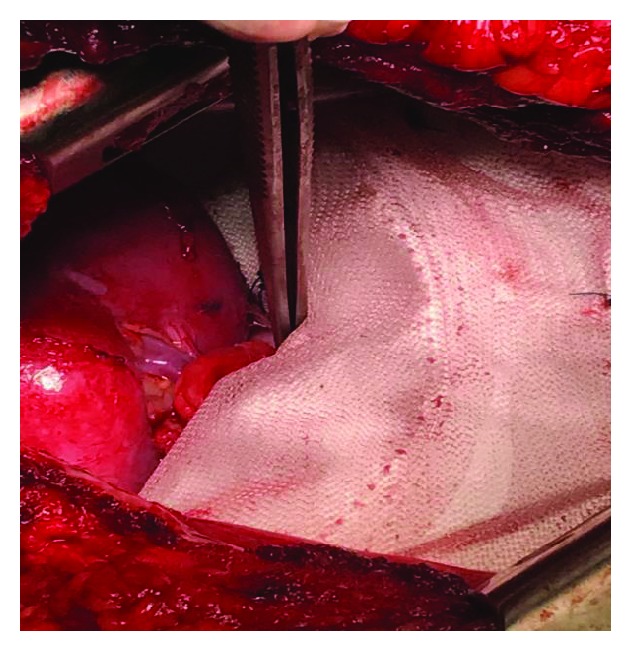
Mesh placement on the hemidiaphragm fixed in the anterolateral position. Note that it surrounds the kidney posteriorly since it remained in the intrathoracic position.

**Figure 11:**
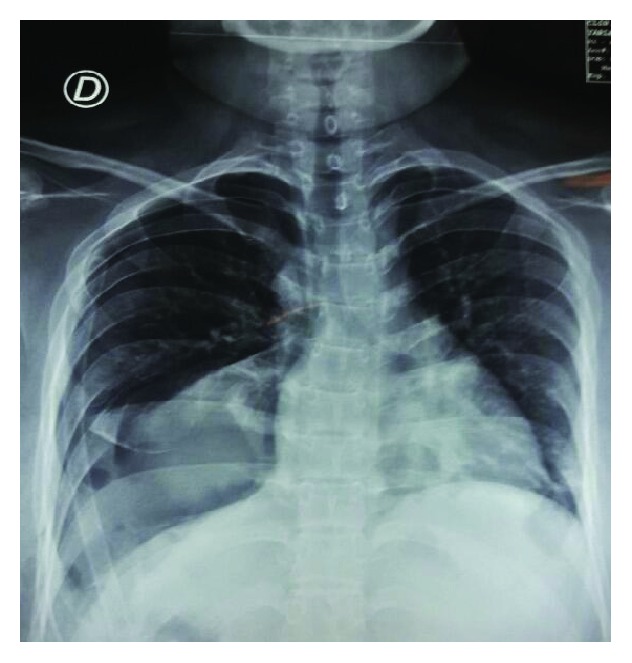
Postoperative radiological view of both hemithoraces, showing right lung expansion, renal silhouette, and the hemidiaphragmatic line. Central trachea and normal left hemithorax.

Laparoscopy revealed visceral displacement, severe elevation of the diaphragmatic dome, and hepatic displacement; there was no diaphragmatic rupture, defined as eventration. The right colon and part of the transverse colon were adhered to the dome. The pedicle of the intrathoracic right kidney ran through the foramen of Bochdalek (Figure 7). A posterolateral thoracotomy was continued at the level of the seventh intercostal space, with the hemidiaphragm being thinned, although with moderate thickness of muscle fibers (Figure 8). An incision was made for hemidiaphragm plication. This was safely performed given the proximity of the peritoneal viscera (Figure 9). It was sutured with prolene 0. A dual mesh of polypropylene-PTFE was placed for reinforcement and fixed at the level of diaphragmatic periphery (Figure 10). There was an improvement of respiratory symptoms. The chest radiograph showed expansion of the hemidiaphragm, pulmonary field, and residual renal silhouette in the intrathoracic position (Figure 11). The patient stayed in the hospital for 12 days with an adequate evolution of the respiratory symptomatology and the operative wound. The pleural effusion through the chest tube was high and remained constant in the first 4 weeks and decreased 2 weeks later, after which we had removed the thoracic tube. This drainage was probably due to the presence of foreign bodies (mesh) that led us to administer octreotide to the patient.

We obtained the patient's written consent for the publication of this case.

## 3. Discussion

Diaphragmatic eventration was first identified by Jean Louis Petit in 1774, the term was first used by Beclard in 1829, and the first surgical repair was described in 1923 by Morrison [2, 4, 11]. Unlike hernia (Bochdalek or Morgagni), there is no disruption of the pleural or peritoneal membrane; however, it is considered a “defect” since anomalous elevation allows invasion of the intraperitoneal viscera into the thoracic space. According to Christensen [2], diaphragmatic eventration is either unilateral or bilateral and complete or partial. Partial presentation is frequent in the anteromedial region of the right hemidiaphragm, whereas complete presentation is commonly found in the left hemidiaphragm [12].

The pathology shows a translucent membrane of up to almost the normal muscle thickness, with the 3 layers of its structure and normal diaphragmatic inserts. The high diaphragmatic level varies and is not a specific criterion to define the condition. The result is decreased respiratory mobility or paradoxical movement, causing poor lung expansion and hypoxia [13]. The prevalence is 5 per 10,000 births (<0.05%), mostly affecting the male sex, and it is frequent in the left hemidiaphragm [10]. It is a relatively rare anomaly in adults [14].

Groth and Andrade [3] differentiated the congenital etiology of true eventration from the acquired one. The embryological changes involve abnormal migration of myoblasts from the upper cervical somites to the transverse septum (4 weeks of gestation) and pleuroperitoneal membrane (8 to 12 weeks); microscopy shows diffused fibroelastic changes and lack of fibers. Eventration is detected at birth or later according to its symptoms and is associated with prematurity, chromosomal anomalies, and developmental defects [15, 16].

Paresis or diaphragmatic paralysis is an acquired condition resulting from certain abnormalities that affect the neuromuscular axis between the cervical spinal cord and the diaphragm. This induces a progressive amyotrophy and stretching of all muscular fibers of the dome. Phrenic nerve injury is one of the frequent causes, especially at delivery, followed by neoplastic infiltration, compressions, or surgical interventions [4, 17, 18]. The lesion of this nerve can be a consequence of neurological pathologies such as myelitis, encephalitis of diverse viral origins, postherpetic neuralgia, polio, tetanus, or diphtheria [4, 10].

The present case was a woman of 17 years of age, with infrequent presentation of complete right defect [12]. The diaphragm presented thickness with muscle fibers, which suggested a normal embryonic development and later perinatal traumatic injury of the phrenic nerve. However, it is important to relate the presence of the intrathoracic kidney, which can cause eventration. The etiology of renal ectopia can encompass infectious problems such as schistosomiasis or pathologies prevalent in northern Peru, such as malaria [9, 19].

Diaphragmatic eventration causes loss of compliance of the chest wall and lack of the normal caudal movement of the diaphragm, which are necessary for inspiration, thus altering the ventilation/perfusion ratio. In adults, it is asymptomatic or associated with respiratory symptoms, such as progressive dyspnea, orthopnea, chest pain, palpitations, or severe respiratory distress due to cranial displacement of the diaphragm. The high dome without contraction causes pulmonary collapse causing atelectasis, bronchial or parenchymal infections, or alterations in myocardial excitability due to mediastinum deviation [20]. In infants, the symptoms are severe due to the poor development of the thoracic cage and weakness of the intercostal muscles that cause paradoxical breathing and require the use of mechanical ventilation [3, 13]. Our patient was treated chronically for bronchial asthma, with episodes of seizures and recurrent bronchial infections. Digestive symptoms, such as nausea, vomiting, flatulence, abdominal pain, epigastralgia, constipation, anorexia, difficulty in gaining weight, and varying degrees of gastroesophageal reflux, may coexist [21]. Pulmonary function tests (PFTs) are important for a dyspneic patient with an elevated hemidiaphragm for highlighting the forced vital capacity and forced expiratory volume in 1 second, showing a restrictive pattern. It is useful to perform the tests in the standing and supine positions. In the supine position, the volumes can decrease to up to 20–50%; nevertheless, these findings do not correlate with the severity of dyspnea, and the tests are mainly used after surgical treatment [3, 17].

The “sniff” test aims at differentiating the actual eventration (congenital) from diaphragmatic paralysis; it is conducted under fluoroscopy, showing a cranial paradoxical movement (PM) instead of a caudal one when sighing. It may not be very reliable because both conditions may involve PM, even in 6% of the normal subjects. Additionally, an eventrated or paralyzed hemidiaphragm may have very little or no PM. It is considered for above 2 cm of PM [17].

A chest radiograph shows the elevation of the affected hemidiaphragm; a shadow of the left eventrated hemidiaphragm should be at least one intercostal space raised with respect to the right and 2 intercostal spaces if it is the opposite side [13]. Foci of pulmonary condensation, atelectasis, or mediastinal deviations can be observed. Bowel loops can be seen in the thorax if a radiopaque contrast agent is used [10]. Ultrasonography has also been used to visualize the PM of the diaphragm. Both chest radiography and ultrasonography are not useful in the differentiation of eventration and diaphragmatic hernia.

Computed tomography and magnetic resonance imaging can be used to accurately determine the elevation of the dome, the viscera in the intrathoracic position, and associated injuries; tumors can be seen in the base of the pulmonary, cervical, or renal ectopia, as in the present case. Reconstructions in coronal and sagittal planes are useful [14, 22]. It is possible to show uninterrupted continuity of the diaphragm to differentiate eventration from hernia, a fact that determines the surgical criteria in case the patient is asymptomatic. Our patient presented this diaphragmatic continuity on computed tomography, which was initially overlooked and required laparoscopic intraoperative detection, as described by Mantoo and Mak [22].

In asymptomatic patients, there are no data comparing the surgical treatment and the conservative approach or the adequate surgical time from the onset of symptoms, especially in the group with phrenic nerve injury; patients with phrenic injury after cardiac surgery may show improvement in the first or second year. Each surgical team establishes its own criteria for surgery. The postsurgery improvement encourages its selection in the majority of patients [23], especially in children, taking into account the lung growth that occurs up to about 10 years. Extreme caution is necessary in patients with morbid obesity or who have neuromuscular disorders [3].

Surgical approaches for eventration include thoracotomy, laparotomy, and thoracoscopy or laparoscopy [1, 11]. Total intravenous anesthesia is preferred due to lower cardiovascular and respiratory compromise and minimal effect on hypoxic pulmonary vasoconstriction [24]. The surgical treatment pursues the improvement of ventilation by minimizing dysfunctional diaphragmatic excursion in the process of inspiration [3]; Yalcinkaya et al. [6] achieved this improvement in 97% of the cases. Plication of the diaphragm [3, 4, 17] improves respiratory mechanics by increasing tidal volume and maximal respiratory capacity. This results in the immobilization of the plicated diaphragm and reduction of the paradoxical movement and contralateral mediastinal involvement [25]. Plication by posterolateral thoracotomy is achieved through the sixth, seventh, or eighth intercostal space, using U points, mattress stitch, continuous suture, or stapling [3]. Resection of the redundant diaphragm and reapproach with overlapping edges have been described, similar to the Mayo technique for umbilical hernia.

Evman et al. [21] reported 42 patients with accordion plication, consisting of several rows of nonabsorbable suture, which are then adjusted to cause “wrinkling” of the diaphragm. The author compared this procedure with the “Mayo technique” that forces the diaphragm to impinge and imbricate the two sheets (“double breasted”). The incision of the diaphragm allows avoiding an intra-abdominal viscera injury below the diaphragm by direct vision. It is advisable not to incise the diaphragm, but there are situations in which it can be done safely and situations in which it is required, such as visceral adhesions under the diaphragm, as per Shah et al. [11].

Thoracoscopy can be performed with two ports and minimum thoracotomy (GelPort) or using three or four ports; the detailed procedure is described by de Andrade Cordeiro et al. [13]. Yalcinkaya et al. [6] successfully reported on 36 patients who underwent thoracoscopic plication with less pain, shorter stay, shorter operative time, and even less cost, resulting in a better pulmonary compliance of the patient in the postoperative period. It is useful in situations such as pregnancy as it is associated with less morbidity [5]. The disadvantage is the reduction of the operative field, although in a recently published case, a thoracoscopic uniportal access was used for the plication with the help of Endostitch [26].

Laparoscopy is associated with less pain as it avoids intercostal nerves; both lungs are ventilated for a better visualization and amplitude for manipulation at the time of suturing. Laparoscopic plication is carried out from the posterior to the anterior position and then from the medial to the lateral, of which a detailed description is made by Groth and Andrade [3, 17]. The prosthetic mesh is used in cases of extreme amyotrophy [4] by placing it on the surface of the muscle, fixing it in the peripheral diaphragmatic inserts, and preventing recurrence at the ends of the plication; some authors are reluctant to use it due to the possibility of infection and increased cost of surgery, although these criteria are relative, if properly managed. Clifton and Wulkan [27] reports the use of PTFE in patients with hernias and eventrations with an excellent description of the operative technique in neonates.

The success of plication clinically improves dyspnea, with favorable changes in PFTs of up to 20% according to Evman et al. [21] and Özkan et al. [28] and a favorable follow-up, which is then maintained for up to 2 years. The image shows the abolition of the paradoxical movement, and although the diaphragm can remain motionless, there is no recurrence of symptoms [25]. Plication reports complications with low incidence, pleural effusions, compartment syndrome, and venous thrombosis.

In the present case, the laparoscopic approach was used for diagnosing eventration and not diaphragmatic hernia; it also helped in the partial reduction of intestinal loops and release of adhesions of the colon in the roof of the hemidiaphragm. The anteroinferior costal arches, pneumoperitoneum, severe elevation of the dome, ergonomic aspect, lack of precision, and lack of experience did not allow suturing in this way. We proceeded by performing posterolateral thoracotomy at the level of the seventh intercostal space, with the hemidiaphragm incision to visualize the peritoneal viscera and avoid injury. Plication was performed by interposing both incised leaves, causing an adequate tension of the hemidiaphragm (“double breasted”). A dual mesh was interposed to reinforce the suture by fixing prolene 0 in the periphery.

In the general population, the frequency of ectopy is reported to range from 0.02 to 0.2% [19]. Intrathoracic renal ectopia accounts for less than 5% of all ectopias. This finding is usually incidental, and the first case diagnosed was reported in 1940 by Wolfromm in a woman aged 42 years. Since then, about 200 cases have been reported [7]. This occurrence is described as frequent in the male sex (63%) and on the left side (62%) [8, 16, 29]. The incidence of intrathoracic kidneys associated with diaphragmatic defects is 0.25% of all ectopias. Ptister-Goedeke and Brunier described four types of thoracic ectopic kidneys: with normal diaphragm, with diaphragmatic eventration, with diaphragmatic hernia, and with traumatic rupture [30]. Our case is not common, as it refers to an adult woman with a right thoracic kidney and is associated with diaphragmatic eventration (type 2).

The etiopathogenesis involves folic acid or vitamin A deficiency in the intrauterine period, exposure to teratogenic drugs, and infections or infestations (schistosomiasis and malaria) or radiation [9]. The criteria to define a congenital chest kidney involve rotational anomaly, long ureter, abnormally high vessels, and deviation of the lower pole [16].

The embryological mechanism is not clear. According to the most accepted theory, the kidney reaches the adult location at the eighth gestational week followed by the superior accelerated migration of the metanephros and the delay in mesonephros involution before completing the diaphragmatic development, that is, before the fusion of the pleuroperitoneal membrane. The persistence of the nephrogenic cord or effects of the liver and adrenal gland development are also described in the pathogenesis [7–9, 16, 29–33]. None of these mechanisms can explain the alteration by itself. Many genes are related to renal anomalies, but it is not known if there is a genetic anomaly that relates the renal ectopia and diaphragmatic defect; however, certain genes (Osr) may be related [29].

The diagnosis of thoracic renal ectopia usually occurs in adults. However, as a consequence of systematic ultrasonographic screenings in pregnancy, precision and diagnostic accuracy, and the use of Doppler, we have diagnostic reports of the condition in the prenatal stage [34], and these have been studied by magnetic resonance imaging as well. The cases reported previously have been associated with congenital diaphragmatic hernia but have not been associated with diaphragmatic eventration, as seen in our case. These reports also confirm the normal functionality of the kidney and suggest that in the absence of diaphragmatic defects, surgery would be unnecessary.

The thoracic kidney is usually asymptomatic and discovered by directed search in adult patients with the absence of the kidney on ultrasonography. The symptomatology is related when there is a diaphragmatic defect, as in the current case, or when the kidney presents lithiasis, obstruction, or tumor. Chest radiography, ultrasonography, intravenous pyelography, computed tomography, and scintigraphy are useful diagnostic tests [7]; other pathologies may show similar findings; therefore, in many cases, a combination of these tests is necessary to clarify differential chest masses, such as neuroblastoma, ganglioneuroma, neurogenic cyst, meningeal cyst, or Bochdalek hernia [9].

Similar to the previous findings, the computed tomography images in this case revealed the kidney to be located in the posterior mediastinum with a complete normal rotational process. It was not found in the pleural space; therefore, there was no pneumothorax. The renal vasculature and the ureter entered and exited the thorax through the foramen of Bochdalek. The ureter was long to reach the excessive bladder distance but did not enter ectopically [29, 35]. No manipulation or treatment is necessary for asymptomatic thoracic kidneys, except when there are other pathologies (vesicoureteral reflux or obstruction) [32], such as those found in this patient.

In conclusion, symptomatic diaphragmatic eventration associated with thoracic renal ectopia is a very rare entity, considering the age, sex, and right location of the anomaly. It represents a clinical and diagnostic challenge; clinicians, radiologists, and surgeons must be alert with a high degree of suspicion to define details and try to understand the etiopathogenesis; in addition, they should plan an adequate and individualized surgical repair making use, as far as possible, of the minimally invasive procedures that are currently used.

## References

[1] Saroj S. K., Kumar S., Afaque Y., Bhartia A. K., Bhartia V. K. (2016). Laparoscopic repair of congenital diaphragmatic hernia in adults. *Minimally Invasive Surgery*.

[2] Christensen P. (1959). Eventration of the diaphragm. *Thorax*.

[3] Groth S. S., Andrade R. S. (2010). Diaphragm plication for eventration or paralysis: a review of the literature. *The Annals of Thoracic Surgery*.

[4] Le Pimpec-Barthes F., Brian E., Vlas C. (2010). Le traitement chirurgical des éventrations et paralysies diaphragmatiques. *Revue des Maladies Respiratoires*.

[5] Rajkumar J. S., Ganesh D., Rajkumar A., Syed A., Guru V. (2017). Thoracoscopic diaphragmatic plication for eventration in pregnant woman: a case report. *Journal of Clinical and Diagnostic Research*.

[6] Yalcinkaya I., Evman S., Lacin T., Alpay L., Kupeli M., Ocakcioglu I. (2017). Video-assisted minimally invasive diaphragmatic plication: feasibility of a recognized procedure through an uncharacteristic hybrid approach. *Surgical Endoscopy*.

[7] Natarajan A., Agrawal A., Purandare N., Shah S., Rangarajan V. (2016). Rare case of thoracic kidney detected by renal scintigraphy. *Indian Journal of Nuclear Medicine*.

[8] Al-Saqladi A. W. M., Akares S. A. (2015). Intrathoracic kidney in a child with literature review. *Saudi Journal of Kidney Diseases and Transplantation*.

[9] Arslan H., Aydogan C., Örcen C., Gönüllü E. (2016). A rare case: congenital thoracic ectopic kidney with diaphragmatic eventration. *The Journal of the Pakistan Medical Association*.

[10] Ozturk O., Yıldız Y., Igde M., Oksuz B. G. (2015). Coexistence of diaphragm eventration and thoracic ectopic kidney. *Hong Kong Journal of Paediatrics*.

[11] Shah S. Z. A., Khan S. A., Bilal A. (2014). Eventration of diaphragm in adults: eleven years experience. *Journal of Ayub Medical College Abbottabad*.

[12] Bharadwaj P. V. V., Reddy V. V. R., Behra G., Praveen J. V. (2014). Right-sided diaphragmatic eventration: a rare entity. *IOSR Journal of Dental and Medical Sciences*.

[13] de Andrade Cordeiro J., Almeida A. K., de Oliveira Júnior S. A., Fernandes B. M., Rego A. C. M., Araújo-Filho I. (2016). Diaphragmatic eventration: review of current knowledge, diagnostic, and management options. *International Journal of Medical Research & Health Sciences*.

[14] Shen C., Che G. (2012). Congenital eventration of hemidiaphragm in an adult. *The Annals of Thoracic Surgery*.

[15] Kulkarni M. L., Sneharoopa B., Vani H. N., Nawaz S., Kannan B., Kulkarni P. M. (2007). Eventration of the diaphragm and associations. *Indian Journal of Pediatrics*.

[16] Sahinoglu Z., Yuksel A., Uludogan M., Bilgic R., Toksoy G. (2011). Left diaphragmatic eventration associated with ipsilateral pulmonary sequestration and intrathoracic kidney in a fetus: reviewing the prenatal diagnosis and etiopathogenesis. *Fetal and Pediatric Pathology*.

[17] Groth S. S., Andrade R. S. (2009). Diaphragmatic eventration. *Thoracic Surgery Clinics*.

[18] Srikrishna M. R., Ravishankar N., Raghavan J., Natarajan M. K. (2010). Surgical repair of congenital diaphragmatic eventration in a septugerian lady. *Revista Brasileira de Cirurgia Cardiovascular*.

[19] Magak P., King C. H., Ireri E., Kadzo H., Ouma J. H., Muchiri E. M. (2004). High prevalence of ectopic kidney in Coast Province, Kenya. *Tropical Medicine and International Health*.

[20] Lau G. T. E., To A. C. Y. (2016). Eventration of the right hemidiaphragm with resultant right atrial compression—a rare finding. *Echocardiography*.

[21] Evman S., Tezel C., Vayvada M. (2016). Comparison of mid-term clinical outcomes of different surgical approaches in symptomatic diaphragmatic eventration. *Annals of Thoracic and Cardiovascular Surgery*.

[22] Mantoo S. K., Mak K. (2007). Congenital diaphragmatic eventration in an adult: a diagnostic dilemma. *Singapore Medical Journal*.

[23] Tsugawa C., Kimura K., Nishijima E., Muraji T., Yamaguchi M. (1997). Diaphragmatic eventration in infants and children: is conservative treatment justified?. *Journal of Pediatric Surgery*.

[24] Rehman A., Mirza Z. A., Yousuf S., Salam A. A. (2015). Anaesthetic management of an adult patient with diaphragmatic eventration. *Journal of Anesthesia & Clinical Research*.

[25] Tiryaki T., Livanelioğlu Z., Atayurt H. (2006). Eventration of the diaphragm. *Asian Journal of Surgery*.

[26] Stamenovic D. (2017). New technique of diaphragmatic plication by means of uniportal video-assisted thoracoscopic surgery. *Interactive Cardiovascular and Thoracic Surgery*.

[27] Clifton M. S., Wulkan M. L. (2017). Congenital diaphragmatic hernia and diaphragmatic eventration. *Clinics in Perinatology*.

[28] Özkan S., Yazici Ü., Aydin E., Karaoǧlanoǧlu N. (2016). Is surgical plication necessary in diaphragm eventration?. *Asian Journal of Surgery*.

[29] Rattan K. N., Rohilla S., Narang R., Rattan S. K., Maggu S., Dhaulakhandi D. B. (2009). Thoracic kidney associated with congenital diaphragmatic hernia. *Congenital Anomalies*.

[30] Yang E. J., Jeong Y. J., Hwang P. H., Lee D. Y., Kim M. S. (2014). Congenital thoracic ectopic kidney associated with diaphragmatic hernia in a 15-month-old boy. *Journal of the Korean Society of Pediatric Nephrology*.

[31] Fiaschetti V., Velari L., Gaspari E., Mastrangeli R., Simonetti G. (2010). Adult intra-thoracic kidney: a case report of Bochdalek hernia. *Case Reports in Medicine*.

[32] Gupta A., Maheshwarappa R. P., Jangid H., Meena M. L. (2013). Ectopic intrathoracic kidney: a case report and literature review. *Hong Kong Journal of Nephrology*.

[33] Sözübir S., Demir H., Ekingen G., Güvenç B. H. (2005). Ectopic thoracic kidney in a child with congenital diaphragmatic hernia. *European Journal of Pediatric Surgery*.

[34] Athanasiadis A. P., Zafrakas M., Arnaoutoglou C., Karavida A., Papasozomenou P., Tarlatzis B. C. (2011). Prenatal diagnosis of thoracic kidney in the 2nd trimester with delayed manifestation of associated diaphragmatic hernia. *Journal of Clinical Ultrasound*.

[35] Madani A., Ghassemi K., Ataei N. (2006). Thoracic ectopic kidney with diaphragmatic hernia. *Tanaffos*.

